# Demand and access to mental health services: a qualitative formative study in Nepal

**DOI:** 10.1186/1472-698X-14-22

**Published:** 2014-08-02

**Authors:** Natassia F Brenman, Nagendra P Luitel, Sumaya Mall, Mark J D Jordans

**Affiliations:** 1 HealthNet TPO, Amsterdam, the Netherlands; 2 Transcultural Psychosocial Organization (TPO), Baluwatar Kathmandu, Nepal; 3 Alan J Flisher Centre for Public Mental Health, Department of Psychiatry and Mental Health, University of Cape Town, Cape Town, South Africa; 4 Centre for Global Mental Health, King’s College London, London, UK

**Keywords:** Demand, Access, Mental health care, Stigma, Treatment gap, Nepal

## Abstract

**Background:**

Nepal is experiencing a significant ‘treatment gap’ in mental health care. People with mental disorders do not always receive appropriate treatment due to a range of structural and individual issues, including stigma and poverty. The PRIME (Programme for Improving Mental Health Care) programme has developed a mental health care plan to address this issue in Nepal and four other low and middle income countries. This study aims to inform the development of this comprehensive care plan by investigating the perceptions of stakeholders at different levels of the care system in the district of Chitwan in southern Nepal: health professionals, lay workers and community members. It focuses specifically on issues of demand and access to care, and aims to identify barriers and potential solutions for reaching people with priority mental disorders.

**Methods:**

This qualitative study consisted of key informant interviews (33) and focus group discussions (83 participants in 9 groups) at community and health facility levels. Data were analysed using a framework analysis approach.

**Results:**

As well as pragmatic barriers at the health facility level, mental health stigma and certain cultural norms were found to reduce access and demand for services. Respondents perceived the lack of awareness about mental health problems to be a major problem underlying this, even among those with high levels of education or status. They proposed strategies to improve awareness, such as channelling education through trusted and respected community figures, and responding to the need for openness or privacy in educational programmes, depending on the issue at hand. Adapting to local perceptions of stigmatised treatments emerged as another key strategy to improve demand.

**Conclusions:**

This study identifies barriers to accessing care in Nepal that reach beyond the health facility and into the social fabric of the community. Stakeholders in PRIME’s integrated care plan advocate strategic awareness raising initiatives to improve the reach of integrated services in this low-income setting.

## Background

Nepal is experiencing a ‘treatment gap’: a discrepancy between people’s mental health needs, and appropriate, accessible care. This ‘treatment gap’ characterizes most Low and Middle Income Countries (LMIC), where the burden of mental illness is substantial compared to higher income countries. There are also resource constraints in these settings making it difficult to detect and treat mental illness [1,2]. The WHO World Mental Health Surveys indicate that the majority of severe cases of mental illness (anxiety, mood, impulse control and substance disorders) in LMIC had received no treatment over the past year [3].

Nepal is a poor country suffering the aftermath of war. This includes an environment where people are at increased risk of depression, anxiety and post-traumatic stress disorder [4]. Poverty, female gender discrimination and caste inequality are also causal factors that contribute to mental health problems such as depression [5]. Less than 1% of the national health budget is presently allocated to mental health [6].

The Ministry of Health is responsible for administering care to the 75 districts of Nepal, through the district health office. One district, Chitwan, is the focus of the present study and is described further in the methods. There is 1 government hospital and 4 private hospitals with psychiatric wards in the district, all of which are located in urban areas. Since the National Health Policy of Nepal was introduced in 1991, there has been an emphasis on decentralizing primary healthcare to the rural population, but due to a lack of resources, mental health care does not reach them. Attention to mental health service delivery is therefore needed at the lowest levels of the healthcare system. Progress has been made implementing this model on a small scale [7] but must be systematically scaled-up within the wider health system [8].

A decentralised approach is essential for narrowing the treatment gap, but will not necessarily ensure that services actually reach those in need of care. Vulnerable people are often marginalised within society and therefore invisible to care providers [9]. Barriers to access and low levels of demand for these services are serious obstacles to meeting the mental health needs of these populations. Factors influencing demand and access to care have received considerable attention within the field of global mental health research, such as the WHO cross-country study into demographic predictors of service utilization [10]. Whilst the burden of mental illness amongst those with a low socioeconomic status in Nepal is high [11], low income also restricts access to mental health care, as people generally have to pay for services themselves. Poverty is therefore a key barrier to accessing care [12,13]. Indeed, higher castes and more educated people are more likely to seek general medical care than any other group [14].

Cultural factors can have an equally powerful influence on service uptake. Attitudes towards health and illness in Nepal are grounded in religious and magical beliefs, particularly in relation to mental health [7]. Help-seeking and healing practices are therefore often centred around traditional healers. The role of healers varies across communities and is currently shifting: often people use both traditional and biomedical sources of care equally effectively [15]. Nevertheless, traditional healers remain very influential and may be a pull factor away from mental health care [7]. Belief systems about mental health in Nepal are complex. Kohrt and Harper [16] argue that divisions between the mind and the body are made in Nepali culture, and that a greater degree of stigma is attached to illnesses of the mind. This has challenging implications for mental health intervention work; failures to systematically apply understanding of such cultural aspects mental health understandings can present a major barrier to demand and access [17,18].

Broadly speaking, mental health stigma is of course not exclusive to Nepal, and is widely recognized as a major barrier to closing the treatment gap across cultural settings. Nevertheless, Clement et al. [19] have only very recently published the first global, systematic review of literature into the impact of such stigma on help-seeking behaviour. In concordance with earlier work [20], they conclude that stigma has a significantly detrimental impact on mental health care seeking, and rates highly in relation to other barriers to care [19]. This review raises the issue of bias towards research conducted in High Income Countries (HIC), with only 8 of 144 reviewed publications were from Asia.

A review focused specifically on mental health stigma towards people in developing countries in Asia [21] did find a comparable effect of stigma on help seeking. However, it raises different context-specific issues in relation to this problem, such as the role of the family, as both ‘stigmatizers’ and ‘victims of stigmatization’; the role of somatization in seeking appropriate care; and the influence of teaching practices on the attitudes of mental health professionals. The small body of research into understanding mental health stigma in Nepal needs building upon [16,22] along with a focus on how this can inform strategic interventions [23]; a need that has been recognised in neighbouring LMIC settings [24]. This study aims to contribute to this goal.

The numerous issues concerning demand and access to mental health care are inextricably linked, but it is useful to delineate between those located at community levels and those focused at the health facility level. James et al. [25] found that the use of services in two LMIC settings (India and Pakistan), were heavily influenced by both the behaviours of community members (‘demand-side factors’ based in the community) and their perceptions of services (‘supply-side factors’ referring to the health facility). Factors anchored in the community were therefore seen to be distinct from those at the health facility, but equally important. This demonstrates the need for an integrated approach to improving the reach of services, which takes into account pre-existing attitudes and behaviours within in the community.

In Nepal, there are research initiatives that explore the best ways to reduce the treatment gap. One such example is the Programme for Improving Mental Health Care (PRIME) [26]. PRIME is a consortium of research institutions and Ministries of Health in five LMIC countries in Asia and Africa, including Nepal. It aims to bridge the treatment gap by integrating mental health care into primary health care [27]. This study is part of the formative research that was carried out to inform the development of a district Mental Health Care Plan. An overview of the complete formative research process has been presented elsewhere [8]. This publication focuses specifically on the themes of demand and access to care, and aims to identify barriers for people with priority mental disorders living in poverty. ‘Priority mental disorders’ (depression, psychosis, alcohol use disorder and epilepsy) are based on the WHO’s mhGAP intervention guide [28]. ‘Poverty’ refers to the social and financial deprivation that we know is associated with poor mental health [29]. We sought to find out how existing services and local attitudes about mental health issues affect levels of demand and access to care, and how we can improve the reach of services in this context.

## Methods

### Study design

This study consisted of individual in-depth key informant interviews (KII) and focus group discussions (FGD). FGDs were used to identify the perceptions of existing groups of stakeholders (such as female community leaders). Individual interviews were necessary to elicit confidential responses to questions about more sensitive mental health related issues. The combination of KII and FGD has been identified as a useful strategy for studying both individual and contextual circumstances surrounding phenomena and to increase the trustworthiness of data [30]. The interviews and focus groups were conducted between April 2012 and September 2012. A team of 4 local research assistants carried out the interviews after receiving 3 weeks training in basic research principles and skills. The interviews were facilitated by an open-ended interview guide, which was developed by the PRIME consortium. This was adapted to focus on key components of the mental health care plan in the Nepali setting. The interviews included an introduction to the research, WHO case vignettes to illustrate the four priority mental disorders (depression, psychosis, alcohol use disorder and epilepsy), and a section focused on demand and access. They were divided into 14 broad questions with multiple probes.

### Setting

Data collection took place in Chitwan, a rural district in the southern, central development region of Nepal. The Maoist People’s War, an intra-state conflict that took place in Nepal between 1996 and 2006, is still taking its toll on the country’s population [31]. Chitwan has slightly better development indicators than other parts of the country, however, and has a higher literacy rate than the national average. Due to migration from all other districts of Nepal, there is a diverse mix of castes and ethnicities in the area, with several different languages spoken. This heterogeneity was one reason the setting was selected for the study. It was also selected for its emergency referral facilities, and the fact that baseline data was available on epidemiological mental health in the area.

Within the healthcare system in Nepal, district hospitals are at the highest level, followed by Primary Health Care Centres (PHCC). 1 PHCC is located in each electoral area, where general medical care is provided. These centres are responsible for monitoring basic health care services at Health Posts (HP) and Sub-Health Posts (SHP): the lowest levels of the network, staffed primarily health assistants, auxiliary health workers, village health workers and auxiliary nurses. In total, there are 31 SHPs, 5 HPs, and 4 PHCCs in the district. Chitwan is comprised of two municipalities and 36 Village Development Committees (VDCs).

### Sampling procedure

The sample was selected from 3 categories of stakeholder: those working at the health organization level, workers at the health facility level and members of the community. A purposive sampling technique, with predefined criteria, was used on all levels and snowball sampling was used at the community level. Inclusion criteria at the health organization level were that national and district level informants were represented. At the health facility level, PHCCs, SHPs and HPs with or without birthing facilities were included. Border sites and both Eastern and Western areas were included to ensure heterogeneity of caste and ethnic groups. Six health facilities were excluded due to their remote locations.

Each respondent was recruited through a home visit or (in case of PHCC workers) a workplace visit. 33 KIIs and 9 FGDs were conducted. A total of 84 respondents participated in FGDs. There was an average of 9 respondents in each group and each group consisted of respondents of a similar educational level and position. All respondents that were approached to take part in the study agreed, and none withdrew their participation. At the end of the data collection period, the total number of KIIs and FGDs were considered to be sufficient for thematic saturation.

### Data collection

Both KIIs and FGDs were conducted in Nepali. Data was audio taped and transcribed directly after fieldwork and each transcript was translated into English for analysis.

### Data analysis

The data were analysed using a Framework Analysis method [32]. Key themes (awareness, demand, detection and identification, and access) were identified *a priori,* to provide a basis for the thematic framework. This was developed throughout the analysis as new themes were identified inductively from the data. The *a priori* themes were based on the strategy for the care package, which was developed over a series of workshops involving healthcare providers, policy makers, health managers and representatives of mental health care organizations and groups.

These strategy development workshops were based on the Theory of Change (ToC) method: an outcomes-based approach for planning and visualising pathways to change [33]. See Jordans et al. for a detailed description of this process [8]. The outcome of these workshops (the final ToC for the care package) was integrated into the final data analysis of this study, so that the findings could be used to fine-tune the package and to inform its implementation.

**Figure 1:**
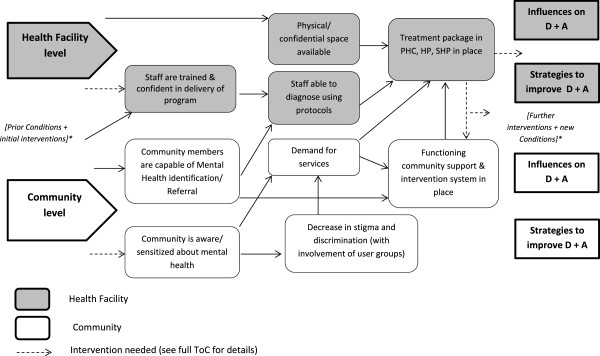
Theory of change map as a framework for data analysis.

Figure 1 shows the section of the ToC that is relevant to demand and access. This illustrates the basic conceptual framework for the findings: *influences* on demand and access at community and health facility levels, and *strategies to improve* demand and access at community and health facility levels. The use of the ToC to guide data analysis is a novel means to link formative research to the design and implementation of such a programme.

Data were coded and analysed from transcripts. A second independent coder from the research team checked the reliability of the thematic framework. All analysis was done using QSR NVivo 9.0 software.

### Ethical issues

Ethical clearance for the study was granted by the Nepal Health Research Council and the Human Research Ethics Committee at the University of Cape Town. The background, procedure and aims of the study were communicated orally to all respondents, along with an assurance that information would be kept confidential and that no payment would be given for participating. Respondents were not identified by name in any transcript, report or publication in order to maintain anonymity. All respondents gave their informed consent in writing, prior to participation.

## Results

### Thematic findings

Table 1 (below) summarises the demographics of the final sample. Table 2 (below) summarizes the main themes relating to both the community and health facility levels that emerged from the data. The text that follows expands upon these themes with direct reference to respondents’ attitudes and perceptions expressed in the interviews and focus group discussions.

**Table 1:** Respondent demographics

**Socio-demographic characterizes**	**KIIs**	**FGDs**	**Total**
**Sex**			
Male	16	30	46
Female	17	54	71
**Age**			
Up to 24	2	1	3
25-59	29	79	108
60+	2	4	6
**Education**			
Literate only/informal education	4	9	13
Secondary level	10	39	49
Intermediate	3	23	26
University	16	13	29
**Religion**			
Hindu	28	77	105
Buddhist	4	5	9
Christian	1	2	3
**Caste/ethnicity**			
Brahmin/chhetri	24	62	86
Janajati	6	12	18
Others	3	10	13
**Occupation**			
Health workers	14	36	50
Senior govt. officer	3	-	3
Female community health volunteers/mothers groups	3	27	30
NGO staff	3	-	3
Others (teachers, traditional healers, political leaders etc.)	10	21	31
Total	33	84	117

**Table 2:** Summary of main themes

	**Factors influencing demand and access**	**Strategies to improve demand and access**
**Health facility Level**	- Service availability: lack of senior staff and insufficient trained village health workers	- Increasing training and resources
	- Mistreatment within health centers	- Building trust in services
		- Protecting family status with increased confidentiality
**Community level**	- Mental health stigma in the wider community	- Reducing the mind-body dichotomy in treatment: working with local notions of stigma around medication specifically for the mind.
	- Low mental health awareness across castes, negatively affecting detection and identification.	- Awareness-raising: Public vs. private information channels
	- Lack of information about services	- Awareness-raising: Trusted and respected figures
	- Conflicting roles of affected families: expected to support access but are in fact barriers.	
	- Cultural norms of visiting traditional healers.	
	- Religious practices (affecting women)	

#### Factors influencing demand for, and access to, mental health care within the community

We present the most salient of the themes extracted from the data. Mental health stigma in the wider community was closely connected to a lack of awareness about mental illness, its effects and its treatment. Poor awareness was a dominant theme throughout interviews and discussions, and was reported to underpin many of the problems relating to demand for and access to services. Although poor and disadvantaged groups inevitably have the lowest levels of awareness, the problem is not exclusive to groups with low socio-economic status. The lack of mental health education in schools means that even those with schooling remain unaware of mental health care,

*"There are still educated people, who believe that it is not mental illness but some kind of spirit possession "bokshilaageko".* KII Auxiliary Nurse Midwife

Furthermore, ignorance about mental health issues was seen to be present amongst those of high economic and caste status. Surprisingly, more education and resources did *not* appear to be associated with being more informed or empathetic towards people with mental illness. In fact, health staff found privileged people to be particularly ignorant and judgemental:

*People involved in politics are uninformed about health and proclaim themselves as big people and label people as mad, do not count them as humans and do not heed their words".* FGD respondent, Auxiliary Nurse Midwife

Many respondents referred the way that people distance themselves from the issue of mental illness. Community members were said to label mentally ill people as "mad" or "crazy" and attribute their behaviour to witchcraft. This in turn reduces their receptiveness to alternative explanations about mental illness. Female Community Health Volunteers (FCHVs) reported a negative response to campaigns against alcohol abuse, saying that people were reluctant to talk to them and were against the campaign. Similarly, Auxiliary Nurse Midwives (ANMs) described their efforts to sensitize their community:

"…*it has been difficult for us to survive if we talk about these things. The community shouts at us not to act so smart and persists calling people with such symptoms as mad and crazy when we tell them not to stigmatize them".* FGD respondent, Auxiliary Nurse Midwife

Poor awareness about such issues has huge implications for the attitudes and behaviour of the community. Attitudes among community members towards those who experience mental illness were often described as extremely negative, involving a combination of denial and hatred. Community members also explained that a lack of understanding of mental illness led to social exclusion and an overall lack of care

*"When the community people, villagers know that so and so person has mental illness then, they do not readily believe them, despise them and mistreat them. Because of this, they hide their problems and it has been difficult to identify their problems".* KII Staff Nurse

*"Some people with mental illness are aggressive, stare blankly "tolaune", shout "jharkine". Moreover, people in return, scolds, accuse them rather than caring them. People do not seek to find out its causes and do not think what they should do to make it better".* KII Auxiliary Nurse Midwife

The above quote illustrates the vicious cycle described by respondents, in which negative attitudes towards mental illness exacerbate problems by forcing sufferers to hide their problems (thus reducing their ability to seek help) and magnifying existing problems (especially if problems relate to depression or social isolation).

Misinformation about services was also commonly mentioned. Not knowing the correct place to seek help was said to reduce demand; some people believe they can only receive treatment from the closest large town. Others are entirely unaware that public health centres exist. This is a particular problem amongst women who are based at home and children from poor families, explaining the perpetuation of low demand in disadvantaged populations:

*"If they are really poor and they cannot go to school, then they do not have knowledge. So they don’t go to PHCC for treatment because they don’t know about it".* KII Teacher

With regard to the influences on demand and access within the family, respondents gave a complex impression of role of family members of people with mental health problems. Respondents often proposed that family members should be responsible for identifying problems, but another common opinion was that the family was responsible for protecting themselves from social stigmatization by hiding the illness:

*Rather than an outsider, [mental health problems] could be identified by a family member of the patient.* KII Health Assistant

*Due to the rumour that [a mental illness] would be healed after marriage, he married a girl and the girl had the same problems, too. When the family knew about it, they hid their son while they exiled their daughter-in-law.* FGD respondent, Female Community Leader

The latter responsibility often manifests in negative reactions towards the family member with mental health problems. Fear of negative family reactions was reported to be a primary cause for potential service users to hide mental illness. Although in general, the family is seen as a source of support for people, this is not commonly the case with mental illness, and in particular, alcohol use disorder. The knowledge that families would withdraw support if they knew about an alcohol problem can make patients highly fearful of sharing this with anyone. This fear is therefore a serious barrier to detection.

*"In case of AUD* (Alcohol Use Disorder)*, if the family used to say that they shouldn’t consume alcohol, then it would be difficult for those people to confess about them being alcoholic. They would try to hide their mistake. The patients are ready to die, but are not ready to expose themselves. The family supports the patient in most of the things, but they do not provide their support in AUD due to which; it would be even more difficult for them to go for treatment".* KII Auxiliary Health Worker

Echoing the problem of mental health ignorance in higher castes, poor detection within families is just as much of a problem in privileged families as it is in poor ones, albeit for different reasons. Whilst poor families cannot afford to send members away for treatment, detection and identification was seen to be difficult in richer communities, because of the threat to family prestige. The expectation that families should support help seeking is therefore problematic, as the quote above indicates that in reality, many people actively hide their problems from their family. Whilst FCHVs called for external actors and professionals to take the lead in this (given the mental health stigma and tight-knit nature of communities in Chitwan), most others believed that this was the role of the family and not the place of an "outsider" (see Health Assistant quote).

Detection, identification and pathways to care (at the community level) are also heavily influenced by cultural and religious influences. Many respondents described spiritual methods of detection and identification as a popular means to identify mental illness. Several respondents referred to the "trend" of looking to the ancestors of the sufferer to seek explanation for symptoms. Spirit possession was reported to characterize many diagnoses amongst certain cultural groups. Respondents described the practice of offering rice seeds to discover the problem of people suffering with mental health complaints.

Social and cultural factors also play an interesting role in determining identification of mental illness. An example was given to illustrate the different cultural meanings of certain behaviours, depending on the person’s caste. A Brahmin, who practices Hinduism and does not drink alcohol, may perceive alcohol consumption as an illness, whereas Gurung, Mangars and Tamangs would perceive this behaviour differently as they all belong to castes in which alcohol consumption is an accepted practice:

*"Cultural beliefs, ethnicity and religious beliefs does make a difference. If someone is drunk and wanders off the street then, the Brahmins call that person an alcoholic while Gurung, Magars and Tamangs say that they must take alcohol as a part of their tradition".* FGD Respondent, Health Assistant

There were some observations of religious practices (Christian and Muslim) explicitly discouraging people from seeking treatment. This was seen to affect women in particular. Muslim women were seen by Female Community Leaders (FCLs) to be restricted in their help-seeking behaviour: they believed the practice of wearing the *burqa* would limit their inclinations to visit the Health Posts. The impact of organized religion was not however discussed at length and help seeking with traditional healers appeared more dominant pathway to care. Indeed, the practice of going to traditional healers over health centres appears a deeply ingrained cultural norm, rather than a conscious choice or direct spiritual demand:

*"Going to traditional healers is something that has been done since ancestral times. If something happens then they go to the traditional healers. No one has to tell them about that- it comes to them automatically".* KII Dhami

The above quote is consistent with the finding that traditional healers are trusted members of the community. Many respondents advocated including traditional healers in the overall mental health system- usually to identify problems and refer to mental health services. Surprisingly however, service users themselves responded much less positively to this, reporting stressful personal experiences, or doubts in the abilities of one or both care providers.

*"Would the traditional healers be able to help us in identification of people with mental illness? I don’t know if they could identify. When I first went to the traditional healers, they said that I was possessed with spirits "lageko" while the doctors said that it was due to excessive tension. Consulting both of them only made me confused and I had no clue whom to believe".* KII Service user

This flags an important point to consider when co-working with traditional healers in this way.

#### Influences on demand and access at the health facility level

The most concerning finding about the health facility was reported mistreatment of mentally ill patients by those in professional positions. The service user respondents did not report experiencing abuse, but some respondents working within health facilities were critical of practices they had witnessed there:

*"Lack of expert mental health human resource and negative perception towards mental health has led the mental health patients to be the victims of worst treatment. They are badly treated during treatment. For example, patients of conversion disorder are sexually harassed and taken advantages. In some cases the health workers and traditional healers have added more problems".* KII Health Assistant

*"Sometimes, the bad behaviour of the people of the health posts would also result in people stopping the treatment".* KII Psychiatric Nurse

This may explain people’s reluctance to seek help from such services or their tendency to abandon treatment from health posts.

Aside from problems of demand or dissatisfaction with services, a simple lack of services and resources in certain areas was found to be a common barrier to care. The lack of human resources was considered to be the most visible and pressing problem.

*"Patients come for treatment but do not find any services".* FGD respondent, Health Assistant

A shortage of staff or training was one reason given for this. The fact that Village Health Workers (VHWs) are not adequately trained means that services are not available where they should be. More senior staff such as Senior Community Medicine Assistants (SCMAs) are often overwhelmed with their workload, and unreachable to some help-seekers. This is particularly detrimental for people living in remote areas, as finding transportation was said to be a serious obstacle. If there is no guarantee that services will be available on arrival, it is unlikely to be considered worth the substantial investment in time and effort to travel to the PHCC.

#### Strategies to improve demand and access at the community level

A suggestion for reducing the stigma attached to seeking treatment for mental illness focused on reducing the dichotomy between treatments for the mind and treatments for the body. One ANM admitted to telling patients that sedative medication was vitamin pills, because of the general mistrust of medication for mental illness. This was not seen as a solution to the problem of low demand, but it was a functional way of encouraging service use, through describing medication in terms of a more holistic or bodily treatment. Another ANM suggested that people would respond more positively if treatment were promoted in more general terms, avoiding too much emphasis on purely mental health problems:

*"Instead of saying that the patient has mental illness if we could tell them that we provide treatment of both problems like difficulty in sleeping, burning sensation in limbs, headache along with mental illness then, more patients will come (R7 agrees)".* FGD respondent, Auxiliary Nurse Midwife

This indicates the importance of considering culturally specific perceptions of different illness. It suggests that people in this community will avoid treatment for illnesses that are presented as specific to the mind, whilst problems that involve both bodily and mental factors are apparently more acceptable to seek help for.

Other strategies focused on awareness raising through either public or private information channels. A vast proportion of responses and discussion focused on the importance of raising awareness and increasing literacy about mental health in communities. There was little doubt that this was one of the most important ways to improve the current situation, however there was less consensus about *the means* by which to carry this out. There was some tension between disseminating information as widely as possible and being sensitive to people’s reluctance to discuss such issues publically. Viewed all together, it appeared that suggestions for public awareness raising, such as television or radio broadcasts, were recommended for providing information about treatment availability and case detection. Mass media (particularly radio) was strongly advocated as a means to disseminate such messages and celebrities were seen as effective awareness-raisers:

*"It would be better if awareness about mental health is raised through Madan Krishna and Haribansha Acharya (famous comedy actors), who played a vital role is spreading awareness about leprosy*". FGD respondent, Auxiliary Nurse Midwife

When it came to communicating a deeper understanding of problems, however, it became clear that people’s sense of taboo and need for privacy would have to be recognized, and respondents gave more cautious suggestions. An example of this is the recommendation from the Mothers Group that education should take place in individual communities or even through home visits, rather than on a mass scale.

The ‘bottom-up’ approaches to attitude change advocated by VHWs and MCHW’s (Mother and Child Health Workers) also fits this model. They felt that direct contact with those who had experienced mental health problems could improve understanding, and again referred to the success of past campaigns in tackling stigmatization against communicable diseases:

*"During the training on HIV, two girls along with a facilitator had come. When we saw those girls a cold chill went down our spine thinking how those cute girls could be infected with HIV. Similarly, for mental health problems too, some service users should be courageous enough to share his/her experience through the training*". FGD respondent, Village Health And Mother And Child Health Workers

A strategy involving the roles and responsibilities for changing attitudes was that awareness raising should be done by two categories of people: those who are *respected* or those who are *trusted* within the community. Respected figures often included celebrities and some mentioned political activists. Those who are respected due to their status within the community were also put forward:

*"Chairperson and secretary of a mother’s group are highly respected and authoritative persons. Various funds collected are all managed under their leadership. Therefore, there is a belief that people support the work initiated by them".* FGD respondent, Female Community Health Volunteer

On a more practical level, trusted and respected traditional healers were also often cited as useful channels of information about service availability, particularly for marginalized people. Similarly, it was suggested that community health workers could act as a bridge for communities who lack trust in external sources of care. Trusted figures also included those who exist within the private sphere, such as sensitized members of mothers’ groups or other community groups. It was thought that known figures were best for community-based awareness raising projects:

*"The people carrying out the projects should be from within the community so that people have more trust upon them and policies can be implemented through them".* FGD, Community Member

#### Strategies to improve demand and access at the health facility level

The most common response to questions about improving detection and identification was that training for health workers is urgently needed. Specifically, respondents called for practical training alongside theoretical courses; prolonged training for HWs and AHWs; and refresher training every few months. Resources needed to improve detection and identification included: protocols for HWs (in the form of pocket guidebooks), books and reading materials for community leaders and health workers, posters for the community, and a referral centre. In the absence of sophisticated resources, respondents stressed the importance of health workers taking a thorough and personalized approach to identifying mental illness (paying attention to family history and building a strong rapport with patients). Facilitating practical means of accessing health facilities (for example by organizing transport) is of primary importance. However, it emerged that the practical accessibility of traditional healers was certainly not the only reason that they were favoured over health facility care,

*"Traditional healers are not present in every village or community. People go to distant places in search of traditional healers".* FGD Respondent, Mothers Group

A more general strategy for improving demand was to build up trust in mental health services. Many saw confidentiality in health facilities as a vital precondition for improving demand. Although openness about mental health was considered important for awareness raising, some respondents were particularly sensitive to people’s need for privacy in this domain.

*There is only one health staff in the health facility for check up. Many people come at once for check up and the patient doesn’t feel comfortable to express his/her problem in front of everyone. Therefore, a separate room is required for check up.* KII Staff Nurse

Suggestions for dealing with this included training health workers to build a sense of trust with patients and creating a separate room (or even a separate centre) for mental health complaints. The promise of confidentiality was one way of protecting family status, when a family member seeks help. This relates the complex role that was identified for the family- as barriers as well as facilitators- to seeking help. Family members were thought to act as barriers when they feared the family would be stigmatized, prompting respondents to suggest protection of status to encourage family support for help-seeking.

## Discussion

The stakeholders involved in this study have provided important insights into the issues that facilitate access and demand for mental health services. This is a crucial aspect of the strategy to integrate mental health care into primary care, above and beyond the actual provision of services. The main findings were that mental health stigma underlies many of the problems regarding demand and access to services at the community level, and that barriers to demand and access at the health facility level are composed mainly of more pragmatic problems. Prejudice and mistreatment towards mental health patients was however also reported to extend to some health workers.

Although the data demonstrate the expected high level of *tangible* barriers to care for marginalized social groups (such as the lack of money and time to travel to health posts [19,20]), they also indicate low demand from all sectors of society, including the more privileged. This appears in contrast to past research on general medical help seeking in Nepal, where higher castes seek more modern medical care [14]*.* This was surprising as we expected that more educated groups would have greater mental health awareness. This was not the case: stigma related barriers to care affected these groups in the absence of poverty related ones, indicating that our initial focus on people living in poverty could be broadened in order to address this issue.

Awareness raising was a recurring theme in responses regarding solutions to identified problems, and was comprised of various means of information dissemination and stigma reduction initiatives. Respondents’ strategies for stigma reduction were consistent with the local literature on this topic [16] and contributed further intervention ideas, which will be valuable for future improvements and scaling up of the package in Nepal. These included the channelling of information through *trusted* and *respected* figures in the community. Traditional healers are well-known sources of such qualities in Nepali society [7]*,* but the findings also pointed to some novel ideas, such as using sensitized members of mothers’ groups to mediate between the health system and those who lack trust in it. Not all strategies were clear cut: the tension between broadcasting information through public channels and more private ones depended very much on the type of education aimed for. These strategies target one of Thornicroft et al.’s three components of stigma: ‘ignorance’ [23]*.* To further this intervention strategy, we could engage in the other components of this model: ‘prejudice’ and ‘discrimination’. Applications of this model are underway in neighbouring India, where evidence based approaches such as social contact have been advocated [24]*.* This method was touched upon by mother and child health workers in this study, who suggested awareness raising through peer education from past service users.

The families and close friends of mentally ill people were considered to be primary detectors and identifiers of problems, and therefore the first step in their relative’s pathway to appropriate care. However, when this is considered in the light of findings about patients hiding mental illness from people close to them (for example, family-in-law), a core problem regarding detection and identification is illuminated: family members and close friends are given responsibility for improving detection and identification of mental health problems, but are *also* recognized as key barriers to this process. This reflects the findings of qualitative research into access for hard- to-reach groups in the UK, which revealed a phenomenon they label the "paradox of demand", where a close social network facilitates access to services, whilst simultaneously disapproving of outside help, creating stigma or providing incomplete information about services [34].

This paradoxical role of the family may well be exacerbated in Asian developing countries, where families have been reported to be particularly likely to feel stigmatized on behalf of a family member [21]. This identifies an obstacle for the PRIME strategy of promoting community detection and identification of mental health problems. Roles and responsibilities will have to be re-evaluated and clarified to ensure this pathway to care is functional. One avenue to explore, generated by respondents themselves, is building a reliable system to protect the confidentiality and therefore the status of patients and families at health facility level.

At the health facility level, problems are generally perceived to be more pragmatic; a lack of trust in the quality and existence of services is a barrier to demand and access.

Overall, respondents suggest that these improvements alone are not to increase service use. Cultural norms and deeply engrained habits of seeking alternative help (i.e. from traditional healers) were portrayed as fundamentally important. This highlights existing influences that interact with those anchored in the health facility to determine the extent to which people will use the care package. This corroborates the findings of James [25]: that the use and effectiveness of mental health care (supply side factors) is influenced by the attitudes and help-seeking behaviours of the local population (demand side factors). Moreover, the finding that mental health stigma also operates in health facilities- through the mistreatment of mental health patients- reminds us that the health facility is embedded in, and inextricably linked to the wider community.

The fact that service users reported doubts about both traditional healers and health workers, after consulting both sources, suggests that this medical pluralism may be more problematic than has previously been reported in this context [15]. This difference may be due to the fact that Shimobiraki and Jimba [15] studied help seeking within general medical care, whilst the present findings pertain specifically to mental health care. Further research into the specific patterns and effects of receiving both traditional and Western mental health care would shed more light on this.

### Implications of the study

Respondents were prolific in their ideas for improving levels of access and demand, identifying underlying problems that required strategic and sometimes conflicting solutions. These data have direct implications for the development of the care package, particularly in increasing the reach of its services. As well as basic awareness raising about the different dimensions of mental illness and its treatment, more holistic stigma reduction initiatives were proposed. Health workers drew on prior knowledge and experience of stigma-reduction within the context of HIV/AIDS or leprosy management, such as peer-education programmes. These are compatible with care models for chronic mental illness that use non-specialized health workers in developing countries [35]. Such suggestions could be followed up by engaging with the broader literature into stigma reduction in Nepal ( [36], for example).

The results also suggested that mental health care should be presented as a part of more general (physical) health care, in order to reduce the role of mental health stigma in creating anxiety about seeking treatment. This implies that implementers could engage with theories about mind-body dichotomies in this culture, in order to reduce stigma. This has been posited as an important issue to understand in global mental health strategies, and has been explored through the aforementioned ethnographic work on mental health care in Nepal [16]. The data certainly reflected these existing findings: people have differential attitudes towards illnesses or treatments that affect the mind, to those that affect the body. The present findings describe pragmatic strategies that health workers adopt and recommend for future interventions, which utilise this understanding.

### Limitations of the study

Although this piece of research captured diverse groups of respondents, which spanned community and health facility levels, a limitation is that it may not represent the most hard-to-reach group that the topic of demand and access is concerned with: those living in extreme poverty or isolation. As part of the large-scale investigation into the functioning of the mental health care package, most respondents in the sample were already engaged with the health systems, either as service users or providers. This meant that it did not necessarily capture the voices of the most marginalized groups. A further limitation is that all transcripts were translated into English for analysis. Although translators were not needed during data collection, words and concepts may have been altered or lost in the translation of text.

## Conclusion

This formative research contributes to the development of a mental health care plan in Nepal. It sheds light on key issues regarding mental health awareness, help-seeking and community detection and identification, in a LMIC that is currently under-researched. The focus on demand and access highlights the barriers to mental health care that reach beyond the health facility and into the social fabric of the community: 1) the lack of trust in services, 2) low mental health awareness 3) high mental health stigma, 4) low detection and identification within families, and 5) cultural and religious norms. Stigma was closely related to family barriers, through processes that have been identified elsewhere in the literature [21,33]. As such, improvements to service provision (including: 1) availability, 2) reliability and 3) confidentiality) at the health facility level, are necessary *but not sufficient* for tackling barriers to care.

Stigma and discrimination should be tackled using approaches advocated by stakeholders. This involves raising awareness about mental health and services through trusted and respected figures, and providing mass awareness raising, whilst remaining sensitive to the needs of community members for confidentiality and privacy. This includes the needs of the families of service users. Such developments will maximize the acceptability of the package for stakeholders, and broaden the reach of its services, as the care plan is scaled-up throughout Nepal.

## Abbreviations

AHW: Auxiliary health worker; ANM: Auxiliary nurse midwife; AUD: Alcohol use disorder; D + A: Demand and access; FCHV: Female community health volunteer; FCL: Female community leader; FGD: Focus group discussion; HP: Health post; KII: Key informant interview; LMIC: Low and middle income country; PHCC: Primary health care centre; PRIME: PRogramme for improving mental health CarE; SCMA: Senior community medical assistant; SHP: Sub health post; ToC: Theory of change; VDC: Village Development Committee; VHW: Village health worker; WHO: World Health Organisation.

## Competing interests

The authors declare that they have no competing interests.

## Authors’ contributions

MJ and NL designed the study. NL coordinated data collection. NB, NL, MJ developed the coding framework, undertook data analysis and wrote the manuscript. SM contributed to the conceptual framework and reviewed the manuscript at several stages. All authors read and approved the final manuscript.

## Pre-publication history

The pre-publication history for this paper can be accessed here:

http://www.biomedcentral.com/1472-698X/14/22/prepub
